# Understanding Critical Quality Attributes for Nanocrystals from Preparation to Delivery

**DOI:** 10.3390/molecules201219851

**Published:** 2015-12-11

**Authors:** Leena Peltonen, Clare Strachan

**Affiliations:** Division of Pharmaceutical Chemistry and Technology, University of Helsinki, P. O. Box 56, Viikinkaari 5 E, 00014, Finland; clare.strachan@helsinki.fi

**Keywords:** bioavailability, drug nanocrystals, permeation, precipitation, solubility, stability, supersaturation

## Abstract

Poor solubility of active pharmaceutical ingredients (APIs) is a great challenge for the pharmaceutical industry and, hence, drug nanocrystals are widely studied as one solution to overcome these solubility problems. Drug nanocrystals have comparatively simple structures which make them attractive for the formulation for poorly soluble drugs, and their capability to improve the dissolution *in vitro* is easily demonstrated, but turning the *in vitro* superior properties of nanocrystals to success *in vivo*, is often demanding: controlled (including enhanced) drug dissolution followed by successful permeation is not guaranteed, if for example, the dissolved drug precipitates before it is absorbed. In this review critical quality attributes related to nanocrystal formulations from production to final product performance *in vivo* are considered. Many important parameters exist, but here physical stability (aggregation tendency and solid state form), solubility properties influencing dissolution and supersaturation, excipient use to promote the maintenance of supersaturation, and finally the fate of nanocrystals *in vivo* are the main subjects of our focus.

## 1. Introduction

Today one of the greatest challenges in drug delivery is the poor solubility of new chemical entities: approximately seventy to ninety percent of new chemical entities are having solubility issues [1]. In the Biopharmaceutics Classification System (BCS) drugs with poor solubility are classified either as class II drugs, exhibiting poor solubility relative to their dose, or as BCS class IV drugs, with both poor solubility and poor permeability. Solubility enhancement improves the bioavailability of BCS class II drugs, but it may also help BCS class IV drugs; since permeation can be enhanced via a higher concentration gradient between the gut and lumen. 

One relatively simple approach to improve drug dissolution and solubility properties is formulation as nanocrystals [2,3,4]. Drug nanocrystals are solid nanosized drug particles surrounded by a stabilizer layer; sometimes they are also referred to as solid micelles. Typically, their size ranges from approximately 200 to 800 nm, depending on the application [5]. The increased dissolution rate is mainly due to the increased specific surface area, but with particle sizes under 1 μm the saturated solubility is also increased (compared to thermodynamic solubility) and the diffusion layer is thinner [4,6].

The selection of suitable stabilizers is crucial for successful nanocrystal formation. Surfactants or polymers, such as polysorbates [7], poloxamers [6], cellulose derivatives [8,9], vitamin E TPGS [8] are all commonly used as stabilizers. In addition, other excipient groups have been investigated for their suitability as stabilizers. For example, Azad *et al.* [10] studied the utilization of superdisintegrants as stabilizers during milling in order to avoid using surfactants, but these are not commonly used. One complicating point with surface-active stabilizers is that they often affect cell membrane functionality. For example polysorbates can make membranes leakier [11] and stimulate P-gp activity [12]. One example of the consequence of this is polysorbate 80 having been observed to increase drug absorption through the sublingual mucosa [11]. Often, stabilizers also increase the solubility of the drug material to some degree.

Though drug nanocrystals are called nanocrystals and are typically indeed crystalline, they may also contain amorphous drug (in part or in whole), depending on the production technique [4]. Liquid-atomization based bottom-up techniques can often precipitate some amorphous material. With other techniques, the product is most often crystalline [3,4], but polymorphic changes may take place with all techniques, during nanocrystallization [13].

*In vitro*, fast dissolution is easily demonstrated with nanocrystalline formulations [14]. However, turning this success into high bioavailability *in vivo*, is often challenging. Physical stability can be problematic; small particles may aggregate, and even more challenging *in vivo* is to hinder the reprecipitation which itself will inhibit drug permeation. We have recently demonstrated this situation with itraconazole nanocrystals in oral drug delivery [15]. Nanocrystal-based itraconazole capsule formulations, which were superior to marketed Sporanox^®^ granules in *in vitro* tests were studied. *In vivo*, the relative bioavailability was only 40% of that of Sporanox^®^. When analyzing the *in vivo* pharmacokinetic profile of the nanocrystal formulations, the dissolution was initially very fast. However, after an initial peak, the concentration rapidly decreased due to precipitation/crystallization. Accordingly, the maintenance of the high supersaturated solution concentration *in vivo* failed. 

The itraconazole case described above is just one example that highlights the importance of controlled drug dissolution followed by successful permeation and underlines the importance of understanding critical quality attributes during the whole lifecycle of the nanocrystal from crystallization to formulation to end product performance. In this article, critical quality attributes related to production as well as final product performance *in vivo* with nanocrystalline products are reviewed. There are many important properties, but perhaps the most important factors affecting the success of nanocrystal products and thus discussed in detail in this review are: (i) physical stability (aggregation tendency and solid state form); (ii) solubility (which itself influences dissolution and supersaturation); and (iii) excipient selection and use together with promotion of supersaturation maintenance. Lastly, the fate of nanocrystals *in vivo* is considered. 

## 2. Properties of Nanocrystals

Characterisation of nanocrystals should consider the interrelated properties of solid state (*i.e.*, crystal form, degree of crystallinity), particle size and morphology, as well as surface/interaction properties (adsorbed stabilizer, surface charge). Table 1 lists some useful characterisation methods, the properties that they probe, as well as the nature of information obtained, and practical aspects of each technique. In the following sections the critical properties of drug nanocrystals, that have been ascertained with these characterization methods, are considered more deeply. 

## 3. Solid State Properties 

The solid state form (polymorphic crystal form, solvate (especially hydrate) form, degree of crystallinity) affects the apparent solubility and hence dissolution rate. Therefore, it is crucial to determine these properties in nanocrystals. Typically, the thermodynamically most stable crystalline form is desirable to prevent the risk of solid state transformations during storage and/or administration [16]. To increase dissolution and bioavailability of nanocrystals, it is possible to prepare the nanocrystals in a metastable crystalline form or even prepare the amorphous equivalent of nanocrystals. However, this is not common in practice.

**Table 1:** Commonly used and more recent methods for characterising nanocrystals.

Category	Characterisation Method	Detection Principle	Information	Data Type	Variations	Sample Requirements	Considerations	References
Solid state form	X-ray powder diffraction (XRPD)	Diffraction of x-rays from lattice planes	Polymorphic form (unique diffraction peaks), amorphous form (no peaks)	Diffractogram, qualitative and quantitative (degree of crystallinity)	Hot stage XRPD to analyse solid state form as a function of temperature	Powder, paste or slurry form, several sample presentation setups possible, amount required depends on setup	Anisotropic particle shape leads to preferred orientation effects (change in relative intensities of diffraction peaks)	[17]
							Peak broadening can occur as crystal lattice size decreases within nanoscale range	
	Differential scanning calorimetry (DSC)	Change in heat flow due to sample changes during heat/cooling	Polymorphic form (melting temperature, crystallisation temperature) amorphous form (glass transition temperature), crystallinity (enthalpy of fusion, enthalpy of crystallisation, heat capacity change at glass transition temperature)	Thermogram, qualitative and quantitative	Modulated temperature DSC to separate overlapping irreversible and reversible thermal events, ultrafast heating	Powder form, few milligrams	Destructive. Results will be different with open or closed (hermetically sealed) pans	[18]
	Infrared (IR) spectroscopy (mid-IR spectroscopy)	Change in dipole moment during molecular vibrations	Polymorphic form (peak shifts and relative intensities), crystallinity (broadening of bands, peak shifts and relative intensities)	Spectrum, qualitative and quantitative, suitable for multivariate analysis	Diffuse reflectance IR (DRIFTS), attenuated total reflection (ATR), microscope	Powder or tablet form, depends on sampling setup, few milligrams. Wet samples usually problematic.	Sample preparation/measurement can involve pressure which can induce solid state transformations	[19,20]
	Raman spectroscopy	Change in polarisability during molecular vibrations	Polymorphic form, crystallinity	Spectrum, qualitative and quantitative, suitable for multivariate analysis	Various sample holders (within spectrometer, sampling probes, microscope)	Powder or suspension, few milligrams (usually). Fluorescent samples are problematic.	Sample heating can be problematic. Samples can be in aqueous medium.	[20,21,22]
Size and morphology	Dynamic light scattering (photon correlation spectroscopy)	Fluctuation of Rayleigh scattering of light associated with Brownian motion of nanoparticles	Particle size, particle size distribution	Particle size distribution (number based mean particle (hydrodynamic) size (Z-average), polydispersity index), quantitative		Suspension with suitable concentration	Suitable only for particles in nanometre size range	[23]
							Viscosity of suspension and temperature affect results	
	Scanning electron microscopy (SEM)	Backscattering of electrons	Topographical information about particles	Scanning electron micrograph, particle morphology, size	Elemental analysis	Dry sample mounted on stage condition setup (vacuum), microgram requirement	Sample preparation destructive	[15]
	Transmission electron microscopy	Transmission of electrons	Density information	Transmission electron micrograph, morphology of cross sections, stabilizer- nanocrystal interaction		Embedded cross section preparation, microgram requirement	Sample preparation destructive	[24]
Surface properties	Zeta-potential	Dynamic electrophoretic mobility under electric field	Surface charge (zeta potential)	Zeta potential, quantitative		Suspension with suitable concentration		[25]
	Surface plasmon resonance (SPR)	Changes in refractive index in the vicinity of a planar sensor surface	Surface adsorption	Spectrum, interaction between stabiliser drug crystals, qualitative and quantitative		Substrate on planar surface sensor required (not direct measurement of nanocrystals)	Careful sample preparation required	[26]
Drug delivery	Dissolution testing	Dissolved drug analysed over time, usually using UV spectroscopy or HPLC	Dissolution profile	Solution concentration vs time	Paddle, flow through cell (with/without membrane insert), pharmacopeial/non pharmacopeial		Separating nanocrystals from dissolution medium can be problematic	[14]
	Fluorescence microscopy	Fluorescence by endogenous or added fluorophores	Localization of nanocrystals in relation to cells and tissues	Fluorescence (and nanocrystal) image	One or two photon (two photon fluorescence offers inherent confocality, sub-micron spatial resolution, deeper penetration in tissues) fluorescence	Non-fluorescent nanocrystals require fluorphore to physically entrapped into nanocrystals	Entrapment and leakage of fluorophore can be difficult or problematic	[17]
	Non-linear Raman microscopy	Change in polarisability during molecular vibrations.	Label free localisation of particles	Intensity of CARS shift (narrow band) or spectrum, (multiplex or broad band). Most commonly qualitative. 2D or 3D images.	Can be dry or aqueous suspension, in cell cultures or tissue samples	Coloured and two-photon fluorescent samples can interfere with signal. Can be coupled with other nonlinear phenomena such as second harmonic generation or two photon electronic fluorescence	Label free. Optimal lateral spatial resolution approximately 300–400 nm.	[21,27]

Different nanocrystal manufacturing methods and conditions can affect the resulting solid state form, and furthermore the thermodynamically stable polymorphic form depends on the environmental conditions. For example, hydrate forms are generally more stable (and therefore less soluble) in aqueous media and in some cases also humid conditions, and therefore if the drug has the potential to form a hydrate form then the potential for conversion should be thoroughly investigated during stability studies in different conditions. X-ray powder diffraction, differential scanning calorimetry and vibrational spectroscopy (infrared and Raman) are the most commonly used methods to establish and monitor the solid state form of nanocrystals (Table 1). 

As mentioned earlier, the solid state properties of nanocrystals are affected by the production method. With bottom-up techniques partial amorphousness is not uncommon, with detrimental effects on the stability of the nanocrystals [28]. Liquid atomization based techniques, like spray drying or electrospraying, are particularly prone to generating a final product in the amorphous form (partially or fully), but full crystallinity can be achieved after production by annealing [29]. The high shear stresses associated with wet ball milling and high pressure homogenization can induce polymorphic changes, but if the milling or homogenization is performed in an aqueous environment the water functions as a plasticizer (raises molecular mobility) and reduces the tendency for sustained formation of amorphous material.

Ali *et al.* prepared hydrocortisone nanosuspensions by both wet-milling and microfluidic nanoprecipitation [28]. With both methods, the particle sizes were approximately 300 nm, yet with milling the product was crystalline, while precipitation resulted in a predominantly amorphous product. In *in vivo* tests with rabbits, the bioavailability during ocular delivery was comparable with both the formulations and when compared to drug solution almost doubled. Differences were clear in stability tests: the crystalline wet-milled nanosuspension was stable for two months (unaltered particle size), but the particle size of the amorphous precipitated nanosuspension had increased to 440 nm.

Lai *et al.* [13] formulated piroxicam nanocrystals with poloxamer 188 as a stabilizer by high pressure homogenization. While the raw material was form I, the resulting nanocrystals were a mixture of monohydrate and form III. The solubility of form I is 14.3 mg/L, while that of form III is 17.0 mg/L. In this case the solubility was increased not only due to the smaller particle size, but also due to the formation of the higher energy solid-state forms.

Pireddu *et al.* studied two different diclofenac sodium crystal forms for (trans)dermal drug delivery [30]. Nanocrystals were produced by wet ball milling, with poloxamer 188 used as a stabilizer. There were no significant differences between the particle size of the two polymorphs when the same milling protocol was used, but differences in the stability of the particle size were seen during 90 days of stability testing. The milling did not change the polymorphic form of the drug. They calculated the crystallite size of the milled polymorphs based on XRPD peak width broadening and found out that for polymorph 1, the crystallite size was around 90 nm while for polymorph 2 it was around 65 nm. *In vitro* penetration and permeation was studied with new born pig skin using Franz diffusion cells. All the nanosuspension formulations improved the drug penetration compared to a commercial gel formulation. Interestingly, though the two polymorphic forms differed in drug permeation properties when administered as coarse suspensions, their nanosuspensions behaved similarly.

## 4. Particle Size and Surface Properties 

**Figure 1:**
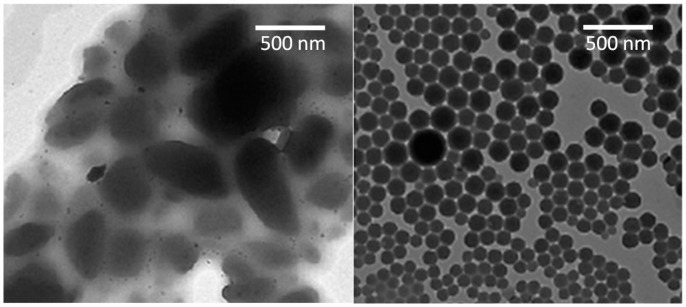
TEM figure of itraconazole nanocrystals produced by nanomilling with poloxamer F 68 as a stabilizer (**left**) and antisolvent precipitation with hydrophobin as a stabilizer (**right**). The compositions and process parameters can be found from the references [7,18], reprinted with permission).

The size, size variation and shape of nanocrystals are related to efficient stabilization of nanosuspensions [31] (Figure 1). The smaller the particle size, the higher the surface energy of the particles, which promotes aggregation. As a result, careful stabilizer selection is crucial when formulating nanocrystals [7,32]. Very rarely, self-stabilization of nanocrystals with any additional stabilizer is possible. This has been demonstrated with 2-devinyl-2(1-hexyloxyethyl)-pyropheophorbide nanocrystals, whose zeta potential, at −40 mV, is sufficient for stabilization [33].

Though the main role of the stabilizers in nanocrystallization is to stabilize the nanoparticles that have formed, most stabilizers in use also enhance permeation to some degree or otherwise influence active transport systems. Accordingly, by careful formulation, not only the dissolution but also the permeation may be actively enhanced with the presence of the stabilizers in nanocrystal based formulations [34].

Most stabilizers are amphiphilic molecules which attach to newly formed drug particle surfaces via hydrophilic-hydrophobic interactions and enhance the wetting of nanocrystals. The classical DLVO-theory describes the stabilization of nanoparticles and the stability can be based on steric and/or electrostatic factors. In most drug nanocrystal studies, stabilization has been based on steric stabilization with, for example, polymers or nonionic long chain surfactants. Steric stabilization can be sensitive to altered temperature. Electrostatic stabilization is reached with ionic polymers or surfactants and if the system is stabilized only by electrostatic forces, the eigenvalue of the zeta potential should be higher than 30 mV. Electrostatic stabilization can be vulnerable if the liquid environment is changed, for example by the addition of ionic species. It is also useful to keep in mind that drying also alters the ionized state, which can itself affect the level of electrostatic stabilization. 

**Figure 2:**
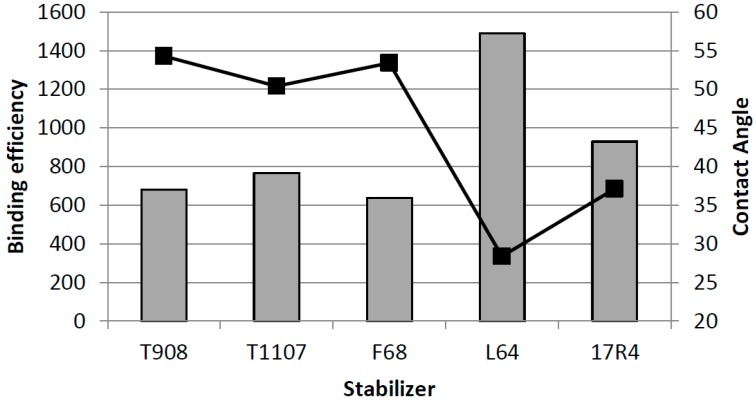
Binding efficiencies of different poloxamers for the indomethacin layers on SPR (grey bars) and contact angle values (black line) for 0.1% (*w*/*v*) stabilizer solutions measured on indomethacin compression surfaces (modified from [26]).

Surface plasmon resonance (SPR) analysis has been utilized in interaction studies between solid drug surfaces and aqueous stabilizer solutions [26]. Five structurally different PPO/PEO block co-polymers were used as stabilizers for indomethacin nanocrystals, and the affinities of stabilizers on solid drug surfaces were determined by SPR and contact angle measurements. Both techniques showed a similar order of efficiency of binding to the solid surfaces (Figure 2). The interaction measurements were compared to successful formation of nanocrystals with the same drug-stabilizer systems by wet ball milling. It was concluded that interaction forces cannot alone determine the most efficient stabilizer, but moderate affinity with longer PEO chains, which are efficient for steric stabilization, formed best nanosuspensions.

## 5. Dissolution of Nanocrystals: Apparent Solubility and Supersaturated State 

Thermodynamic solubility is the solubility of the most stable crystalline form of the drug in a given medium at defined temperature and pressure. Solubility can temporarily be higher than the thermodynamic solubility, which may be observed with, for example higher energy amorphous forms, metastable polymorphic forms, or nanosized drug particles. This elevated solubility has been described with varying terms, such as kinetic or apparent solubility, with the latter term being used in this review. Since the apparent solubility with nanosized particles is higher than the thermodynamic solubility of the material, dissolution of nanocrystalline material is likely to lead to a supersaturated solution, also known as the “spring effect”. 

Ige *et al.* studied the saturation solubility of fenofibrate nanocrystals [35], which had been reduced in size from 80 μm (bulk drug) to 460 nm (nanocrystals). The thermodynamic solubility of the bulk drug in aqueous 0.5% and 1% sodium dodecyl sulfate solution, was 6.02 and 23.54 μg/mL, respectively, while the corresponding values for drug nanocrystals were 67.51 and 107 μg/mL, respectively. 

In another study, the intrinsic dissolution rates and surface concentrations with differently sized indomethacin nanocrystals with two different poloxamer stabilizers were studied [6]. Intrinsic dissolution rates were measured with a channel flow system. The intrinsic dissolution rates were affected by both the particle size and the stabilizer. With the smallest nanocrystals (580 nm), the intrinsic dissolution rate with poloxamer F68 as a stabilizer was 0.50 μg/min/mm^2^, while that for poloxamer F127 was 0.31 μg/min/mm^2^. The dissolution rate of bulk indomethacin was also measured, and was much lower at 0.05 μg/min/mm^2^. Surface concentrations have also been measured with UV-imaging [6]. Again, differences in concentrations were affected by both particle size and stabilizer (Table 2). For example, with equally sized nanocrystals (580 nm), the surface concentration after 10 min of dissolution was 28.7 mg/L with Pluronic F 68 while with Pluronic F127 the corresponding value was 22.1 mg/L. With both stabilizers, the difference with bulk indomethacin was dramatic; the surface concentration with bulk indomethacin was only 2.1 mg/L.

**Table 2:** Surface concentrations after 10 min of dissolution of indomethacin compressed surfaces (modified from [6]).

Sample	Concentration/mg/L		
	Distance from the Dissolution Surface		
	0 mm	2 mm	3 mm
Nanocrystals with Pluronic F 68, particle size 580 nm	28.7	11.7	5.8
Microcrystals with Pluronic F 68	11.4	4.0	2.6
Nanocrystals with Pluronic F127, particle size 580 nm	22.1	9.4	4.3
Microcrystals with Pluronic F127	17.1	7.6	4.0
Bulk indomethacin	2.1	0.3	0.0

As already stated in the Table 1, dissolution and solubility testing of nanocrystals can be demanding due to the separation problems: undissolved nanocrystals should be able to be separated from the dissolution medium before concentration determinations. For that both ultracentrifugation [7] and filtration [26] or their combination [19] has been used. Both methods has their own limitations [14]. In filtering the drug may interact with filter material and/or smallest particles can pass through the filter. On the other hand, in centrifugation due the long centrifugation time and increased temperature drug may be absorbed to tube material and/or also further dissolution may occur during the process [14]. Liu with coworkers [14] studied impact of ultracentrifugation and filtration on the dissolution results. Indomethacin had interactions both with one filter type tested as well as with centrifuge tube material, but it was concluded that by careful selection of filter type the filter was still the best choice in that study. Undissolved drug particles in the sample can be recognized by utilizing more than one wavelength in the analysis. Sarnes with colleagues [6] determined drug concentrations with solubility testing of nanocrystalline samples with UV-spectrophotometer. The drug concentration determinations were done with the wavelength were the drug had its absorption maximum, but the absence of undissolved particles were confirmed with the wavelength were the absorbance of the excipient and drug was negligible. 

Eventually, precipitation may sooner or later occur until the concentration equals the thermodynamic solubility. Furthermore, changes in the composition and pH of the solution such as in the gastrointestinal tract will affect solubility and hence the tendency for crystallisation. In order to improve the bioavailability *in vivo*, the supersaturated state should be maintained and precipitation hindered. Some polymers, such as polyvinyl pyrrolidone (PVP) [36], methacrylate co-polymers [37], hydroxypropyl methylcellulose (HPMC) [38], and hydroxypropyl methylcellulose acetate succinate (HPMC-AS) [38] are effective at maintaining (or at least helping to maintain) supersaturation. This is termed the ‘parachute effect’. Solid dispersions, especially where the amorphous drug is dispersed on a molecular level within the polymeric crystallization inhibitor, are well established as parachute promoters. However, the permeation from supersaturated solutions may be hindered by the precipitation inhibitor, as is the case often with solubilizing agents, when the drug favors the micelles instead of permeation [39]. 

The parachute effect of the polymer can be due to a combination of mechanisms [40,41,42,43]. First, the polymers can themselves increase the thermodynamic solubility of the drug (also known as the co-solvency effect) which reduces supersaturation and consequently the thermodynamic driving force for crystallisation (importantly, this also leads to an additional spring effect with the polymer) [44]. Through drug-polymer complexes in solution via electrostatic bonds, van der Waals’ forces or hydrogen bonding, even the addition of small amounts (0.1%–0.25% *w*/*w*) of polymers such as PVP and HPMC to solution can significantly increase the aqueous solubility [45]. Second, polymers adsorbed on solid surfaces (e.g*.*, with nanocrystals) can block the interaction of already dissolved drug molecules with crystal surfaces and thereby crystal growth. Electrostatic bonds, van der Waals’ forces or hydrogen bonding can all affect the interaction between the polymer and crystal faces, and therefore the degree of crystal growth inhibition. Third, the viscosity of the polymer solution may also inhibit the diffusion of the molecules which limits crystal growth [44].

Ghosh *et al.* [8] formulated nanocrystals from a poorly soluble drug with TPGS or TPGS with a co-stabilizer (HPMC, PVP, poloxamers). During *in vivo* analysis with dogs, the AUC value was nine times higher and *C*_max_ five times higher with nanosuspension than with coarse drug formulations. The physical stability during storage with TPGS alone was considerably lower than for the mixed systems.

Ueda *et al.* [46] studied the maintenance of supersaturation with amorphous and nanocrystalline formulations of carbamazepine. For in depth analysis of supersaturation, they conducted real-time monitoring with NMR spectroscopy of the dissolved carbamazepine with both amorphous and nanocrystalline drug in supersaturated solution. Based on ^1^H-NMR measurements, the dissolved concentrations for nanocrystalline carbamazepine were nearly constant for 50 h. The authors concluded that nanoparticle formation lowered the degree of supersaturation, leading to a relatively stable supersaturated solution of carbamazepine. The presence of nanoparticles also suppressed the formation of large precipitates. With spray dried amorphous carbamazepine, the initial concentration was higher but it then dropped below the concentration of the nanocrystalline sample, indicating that the higher supersaturation was more kinetically unstable with fast precipitation/crystallization of large microparticles. The particle size of nanocrystals in this study was approximately 150 nm.

## 6. Drug Absorption from Nanocrystalline Formulations 

As mentioned above, drug absorption is a function of both solubility and permeability. Solubility is typically negatively correlated to lipophilicity. Dissolution from nanocrystals is followed by permeation of the dissolved drug across the gastrointestinal wall (in the same way as drug from a solution formulation). In addition to increase permeation due to higher dissolved concentrations, stabilizers themselves interact with cells and epithelial cell layers to enhance permeation. However, this requires the simultaneous presence of both drug and stabilizer in the presence of cells, which may not always be the case.

Li with colleagues [47] studied the effect of drug physicochemical properties on oral bioavailability. They studied five different drugs and nanocrystals were produced with the same stabilizer, poloxamer 188, by high pressure homogenization. Particle size with all the tested drugs was from 430 to 460 nm. The AUC values for nanocrystals were in all the cases 1.4–7.2 times higher as compared to drug microsuspensions after oral administration of suspensions to rats. Melting point, log P value and polar surface area affected drug absorption and drugs with low melting point, log P value approximately 5 and polar surface area value between 50 and 60 showed higher absorption with the same sized nanocrystals.

Many stabilizers used for nanocrystal products (vitamin E TPGS, poloxamers, polysorbates) are also P-gp inhibitors [12]. PEG chain length (between 200–6000 Da) in TPGS may affect the inhibition activity and the best inhibition is reached with PEG chain lengths of 1100–1500 Da [48]. 

In some cases, nanocrystals can be taken up by cells (e.g., [21]). This may be desirable (e.g., with cancer cell targeting) or undesirable (unpredictable pharmacokinetic profiles, see below). Uptake will vary between cell types and their phagocytotic/endocytotic potential, as well as nanocrystal properties such as size, morphology, stabilizer type, and surface charge. The wide selection of possible nanocrystal formulations, and potential importance highlights the need, in some cases, for an understanding of the nanocrystal behaviour on the cellular and tissue levels [49]. 

Chen and Li [34] studied the cellular uptake mechanism of paclitaxel nanocrystals. They found out that nanocrystals were internalized by KB cells with higher concentrations than solubilized formulations. They also concluded that drug nanocrystals were possible to take up as solid particles probably via endocytosis. However, the surface layer of the nanocrystals affected the uptake. Based on confocal imaging and temperature dependent internalization it was concluded that endocytosis is probably responsible for the nanocrystal uptake by cells. Accordingly, nanocrystalline chemotherapeutic formulation can possibly form intracellularly lethal microenvironment for the cell when the drug nanocrystals are slowly dissolved inside the cells. This can be difficult to reach with solubilized drug delivery systems. The mean particle size in this study was from 230 nm to 280 nm.

The ability of TPGS stabilized paclitaxel nanocrystals to reverse P-glycoprotein drug-resistance in P-gp overexpressing H460 cancer cells was evaluated by Gao with colleagues [50]. They found out that TPGS as a stabilizer on paclitaxel nanocrystals efficiently reduced drug resistance of the studied cells. It is known that due to the EPR effect, drug nanoparticles can accumulate after intravenous injection in the tumor tissues [51]. But therapeutic efficacy is limited by overexpressing MDR related proteins like P-gp in resistant tumours. It has been shown that nanosized materials can be taken up via endocytosis by cells, but after dissolution into cellular cytoplasm they can be pumped out by P-gp efflux system [52]. Hence, utilization of simultaneous lowering of P-gp activity with endocytosis of nanoparticles can increase the therapeutic efficiency, like was the case with TPGS coated paclitaxel nanocrystals [50]. Drug and P-gp inhibitor should be at the same time inside the cells. This was not realized when TPGS was given in solution together with free paclitaxel molecules [53], but with TPGS coated paclitaxel nanocrystals it worked [50].

While electron microscopy and fluorescence imaging are the two main established methods to image the physical interaction of nanoparticles with cells [49,54], including their uptake and localization within the cells they have some drawbacks (e.g., lack of chemical specificity and inability to probe live cells with electron microscopy), and complications with fluorescent labels, including label leaching and overestimation of nanocrystal internalisation when the fluorescent labels but not necessarily the nanocrystals themselves enter the cells [55,56]. Thus, it is worth considering novel analytical techniques in this context. 

Confocal Raman microscopy and coherent anti-Stokes Raman scattering (CARS) microscopy are relatively novel label-free, chemically specific and non-destructive methods with potential for label-free imaging of nanocrystal-cell interactions. With these techniques submicron particles may be detected provided they have a sufficiently strong Raman or CARS signal (the resolution and speed is better for the inherently confocal CARS technique, while chemical specificity is better for Raman microscopy) [27,57,58].

**Figure 3:**
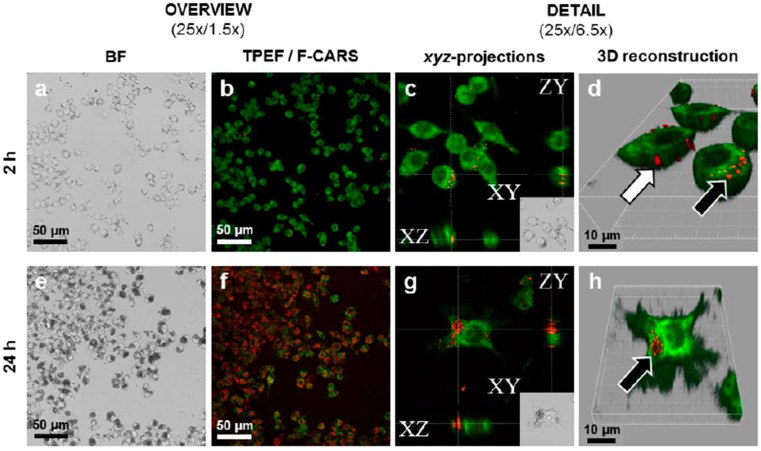
Interactions between paliperidone palmitate (PP) nanocrystals and RAW 264.7 macrophages imaged by CARS after 2 (**a**–**d**) and 24 h (**e**–**h**). Imaged is performed using CARS signal at 2860 cm^−1^ and fluorescently dyed cell membranes are imaged using TPEF. (**a**,**e**) low and high magnification brightfield imaging; (**b**,**f**) forward-CARS (red)/TPEF (green) merged micrographs of stained/fixed cells; In (**c**,**g**) intracellular PP nanocrystals are seen in orthogonal projections of z-stacked F-CARS/TPEF overlays; (**d**,**h**) show 3D-reconstructions of the z-stacked F-CARS/TPEF overlays. White arrows indicate PP-NC adsorbed onto cell surface and black arrows phagocytosed PP-NCs. (From [21], reprinted from Elsevier with permission).

In a proof of concept study that also had clinical relevance [21], Darville *et al.* imaged the fate of nonfluorescent nano/micro crystals of the antipsychotic prodrug, paliperidone palmitate, in macrophage cell cultures and histological sections using CARS microscopy (Figure 3). The commercially available product Xeplion^®^ is a long-acting aqueous suspension for intramuscular injection with a measured median volume based equivalent sphere diameter of approximately 1000 nm and a *D_V_*_,10_ of approximately 450 nm. 

The active species, paliperidone, is esterified with the palmitate moiety to reduce solubility and associated dissolution rate, thereby sustaining the release of the paliperidone. *In vivo*, complex and variable pharmacokinetic profiles have been observed [59] and in rats the formation of granulomatous tissue in the region of the intramuscular nanocrystals has been observed. The inflammatory response led to particle agglomeration, phagocytosis and radial angiogenesis in the rats resulting in multiphasic systemic absorption of the paliperidone being observed [21]. CARS microscopy was used to investigate the fate of the paliperidone palmitate nanocrystals with macrophage cells *in vitro* and histological sections *in situ* in some detail. The nanocrystals were imaged in both fixed and live cells using the CH_2_ stretching resonance at 2845 cm^−1^, mainly associated with the palmitate moiety (the nanocrystals were resolved from endogenous lipid in this case through geometrical differences, and an otherwise weak lipid signal from the cells was used, although with other drugs a CARS resonance resolved from lipid signals could be used for chemical specificity). In tissue sections, intracellular nanocrystals were imaged within the granulomatous tissue.

## 7. Conclusions 

Drug nanocrystals are a highly feasible option for enhancing drug release profiles with poorly soluble drugs. However, in order for nanocrystal formulations to be successful *in vivo*, a thorough understanding of their critical quality attributes from the production step to the end product performance is required. With nanocrystal formulations the physical stability should be monitored carefully. Solubility properties needs to be known as well as their effect on supersaturation. Even more important is the control of precipitation after rapid dissolution and supersaturation *in vivo,* in order to promote fast and efficient permeation. This can be reached for example by utilization of excipients promoting the maintenance of supersaturation in the formulations. Finally, reaching reliable *in vitro in vivo* correlation should be confirmed and understood on a mechanistic level: the high bioavailability of nanocrystal formulations is based on careful formulation design, suitable analysis methods and recognition of the most important critical quality attributes.
